# A Two-Step Method for Obtaining Highly Pure Cas9 Nuclease for Genome Editing, Biophysical, and Structural Studies

**DOI:** 10.3390/mps1020017

**Published:** 2018-05-30

**Authors:** Nandhakishore Rajagopalan, Sateesh Kagale, Pankaj Bhowmik, Halim Song

**Affiliations:** National Research Council of Canada, 110 Gymnasium Place, Saskatoon, SK S7N 0W9, Canada; Sateesh.Kagale@nrc.gc.ca (S.K.); Pankaj.Bhowmik@nrc.gc.ca (P.B.); Halim.Song@nrc.gc.ca (H.S.)

**Keywords:** genome editing, CRISPR-Cas9, ribonucleoprotein (RNP), Cas9 purification, cation exchange chromatography

## Abstract

Cas9 is a site-specific RNA-guided endonuclease (RGEN) that can be used for precise genome editing in various cell types from multiple species. Ribonucleoprotein (RNP) complexes, which contains the Cas9 protein in complex with a guide RNA, are sufficient for the precise editing of genomes in various cells. This DNA-free method is more specific in editing the target sites and there is no integration of foreign DNA into the genome. Also, there are ongoing studies into the interactions of Cas9 protein with modified guide RNAs, as well as structure-activity studies of Cas9 protein and its variants. All these investigations require highly pure Cas9 protein. A single-step metal affinity enrichment yielding impure Cas9 is the most common method of purification described. This is sufficient for many gene editing applications of this protein. However, to obtain Cas9 of higher purity, which might be essential for biophysical characterization, chemical modifications, and structural investigations, laborious multi-step protocols are employed. Here, we describe a two-step Cas9 purification protocol that uses metal affinity enrichment followed by cation exchange chromatography. This simple method can yield a milligram of highly pure Cas9 protein per liter of culture in a single day.

## 1. Introduction

The Clustered Regularly Interspaced Short Palindromic Repeats (CRISPR)/CRISPR-associated (Cas) system is found in bacteria and archaea and act as their adaptive immunity against invading foreign nucleic acids [1]. Due to its targeting simplicity, this system has been rapidly adopted as the method of choice for editing of genomes of multiple organisms. However, the widely used Cas9 nuclease produces modifications at multiple off-target sites leading to concerns about its specificity [2]. Efforts are being made to study the structure–function relationships of the RNA and the nuclease and engineer specific nucleases. Further, chemical modifications to these gene editing components to enable better cell entry or specific targeting to particular tissues are also being carried out. A simple and rapid protocol for obtaining pure nuclease protein is essential for these biophysical, chemical modification, and structural biology efforts. Often, for genome editing techniques and in vitro nuclease assays, Cas9 is purified by a single-step metal affinity enrichment [3,4,5]. However, for higher purity of Cas9 additional purification, steps like cation exchange chromatography and size exclusion chromatography have been added to the initial affinity enrichment [1,6]. We have developed a simple two-step Cas9 purification protocol that yielded milligrams of highly pure protein. We achieved this by adding a high-resolution cation exchange chromatography step right after the affinity enrichment of Cas9. Using this protocol, we were able to purify Cas9 to near homogeneity within a few hours.

## 2. Experimental Design

**Figure 1:**
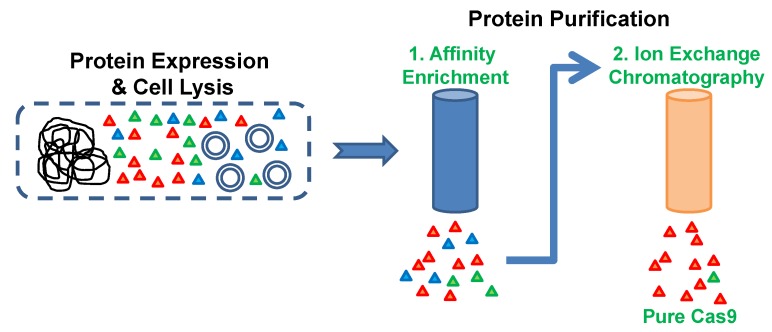
Protein purification scheme. Cas9 protein was expressed in *E. coli* host cells and the harvested cells were lysed by French press. The Cas9 protein having a C-terminal His-Tag was enriched from the soluble cell lysate by using metal affinity chromatography. Cas9-enriched fraction was directly applied to the high-resolution cation exchange chromatography column for obtaining pure Cas9 protein.

The Cas9 purification protocol described here has four parts among which two are protein purification steps (Figure 1). A chromatography system like the ӒKTA FPLC (Fast protein liquid chromatography) system and a high-resolution cation exchange column like the Resource S (6 mL column volume) are essential to perform this purification. We use the French pressure cell for bacterial cell lysis. This is an efficient and mild lysis method that we find very effective for bacterial cells. Others have successfully used sonication to lyse cells for Cas9 purification [4,5,6]. However, caution is required while using sonication and care must be taken not to overheat the samples as it might cause protein denaturation. Varied buffer components and compositions have been employed by different groups to serve different purification and downstream functional requirements. A reducing agent is often added during the cell lysis stage and maintained in the purification as well as the storage buffers. Some protocols have used β-Mercaptoethanol (β-ME) as a reducing agent during cell lysis [4]. Although this is stable, caution must be used while handling it as it is toxic and also has a strong and unpleasant odor. Moreover, β-ME can form adducts with free cysteines in the protein and this has to be taken into account in any subsequent protein analysis like mass spectrometry or chemical modification protocols. Another reducing agent that is used in Cas9 purification is dithiothreitol (DTT) [5]. However, compared to β-ME, DTT is unstable and must be avoided in sensitive techniques like isothermal titration calorimetry (ITC) or structural biology. We and some other groups have used the stable and odor-free reducing agent Tris(2-carboxyethyl)phosphine (TCEP) [1,6]. Further, TCEP would be useful for any downstream chemical modifications of the free thiol groups of cysteine residues, especially with maleimide-linked reagents. Protein purification protocols for unstable proteins call for the use of a cocktail of protease inhibitors. However, we did not find any significant changes in the elution profile or the final yield of Cas9 with the addition of these inhibitors. Hence, we concluded that the additional bands observed below the Cas9 protein band in the affinity purification were not proteolytic products of Cas9. Additional steps are required to obtain highly pure Cas9 after the initial affinity enrichment. Other groups have employed a HiTrap SP column (GE Healthcare) for cation exchange chromatography purification of Cas9 [1,6]. The HiTrap SP columns with their larger bead sizes provide a low-resolution purification and hence we used a high-resolution Resource S column for our final step of purification. This enabled us to obtain higher purity of Cas9 in two purification steps. A general scheme of the purification strategy is provided in Figure 1. 

### 2.1. Materials

- pET-NLS-Cas9-6xHis was a gift from David Liu (Addgene, Cambridge, MA, USA; plasmid no. 62934)
- *Escherichia coli* BL21 star (DE3)pLysS (ThermoFisher Scientific, Waltham, MA, USA; Cat. no.: C602003)
- High vacuum grease (Dow Corning, Midland, MI, USA; Cat. no.: 1597418)
- HEPES (Sigma-Aldrich, St. Louis, MO, USA; Cat. no.: H-3375)
- Potassium chloride (Sigma-Aldrich; Cat. no.: P3911)
- Magnesium chloride hexahydrate (Sigma-Aldrich; Cat. no.: M2670)
- Sodium chloride (Sigma-Aldrich; Cat. no.: S7653)
- Tris(2-carboxyethyl)phosphine hydrochloride (TCEP; Sigma-Aldrich; Cat. no.: C4706)
- Imidazole (Sigma-Aldrich; Cat. no.: I-2399)
- Glycerol (Sigma-Aldrich; Cat. no.: G5516)
- Ni-NTA superflow (Qiagen, Hilden, Germany; Cat. no.: 30430)
- Econo-Pac chromatography columns (Bio-Rad; Hercules, CA, USA; Cat. no.: 7321010)
- Protein assay dye reagent concentrate (Bio-Rad; Hercules, CA, USA; Cat. no.: 5000006)

### 2.2. Equipment

- French pressure cell (35 mL cell with 1” diameter piston; AMINCO, Silver Spring, MD, USA; Cat. no.: 4-3339)
- French pressure cell press (AMINCO, Lake Forest, CA, USA)
- Beckman J2-MI centrifuge (Beckman, Brea, CA, USA)
- Beckman Coulter Ja-17 rotor (Beckman)
- Beckman Coulter Allegra X-15R centrifuge (Beckman)
- Beckman Coulter SX4750 swinging bucket rotor (Beckman)
- Resource S, 6 mL cation exchange column (GE Healthcare, Uppsala, Sweden; Cat. no.: 17-1180-01)
- ӒKTA FPLC system (Amersham Biosciences, Little Chalfont, UK)
- Amicon Ultra-15 centrifugal filters, 50K cutoff (Millipore, Burlington, MA, USA; Cat. no.: UFC905024)
- Spartan-30 HPLC (High-performance liquid chromatography) certified syringe filter with regenerated cellulose membrane, diameter: 30 mm, pore size: 0.2 μM (Whatman, Maidstone, UK; Cat. no.: 10463060).

## 3. Procedure

This protocol describes the cell lysis and purification of NLS-Cas9-6×His protein expressed from the pET-NLS-Cas9-6×His vector [6] in *E. coli* BL21 star (DE3)pLysS cells. The volumes of solutions and materials described in this protocol should be sufficient for the purification of the protein from cells harvested from 2 L of bacterial culture media. Guidelines for transformation of *E. coli* strain with the expression plasmid and expression of recombinant protein can be found in the manufacturer’s manual (e.g., ThermoFisher Scientific Manual part no. 25-0402). Briefly, a 6 L shake flask with 2 L of sterile Luria–Bertani (LB) media containing Ampicillin (100 µg/mL) was inoculated with 5 mL of overnight starter culture of NLS-Cas9-6×His. The flask was incubated at 37 °C until absorbance at 600 nm reached 0.6. Cas9 protein expression was induced by adding isopropyl β-d-1-thiogalactopyranoside (IPTG) (0.5 mM) and incubation at 18 °C for 16 h. Cells were harvested by centrifugation at 5000× *g* for 15 min at 4 °C.

### 3.1. Cell Lysis and Isolation of Soluble Proteins. Time to Completion: 1 h

1. Adjust the volume of harvested cells to 35 mL by adding ice-cold buffer LW (for cell lysis and washing out unbound molecules).
2. Suspend the cell mass in the buffer thoroughly by vortexing and placing the tube in an end-over-end rotation shaker. To prevent clogging of the cell lysis equipment, ensure that there are no cell clumps at the end of this step.
3. Load the cell suspension into a pre-cooled 35 mL French pressure cell equipped with a 1-inch diameter piston. Lubricate the piston and cap of the French pressure cell with high vacuum grease to ensure smooth operation.
4. Close the cell and load it in the cell press device. Set the cell pressure to approximately 16,000 psi and open the key gently to release the suspension and collect the cell lysate in a fresh tube. The key should not be opened wide and the cell pressure should be maintained close to the set pressure.
5. Repeat steps 3 and 4 to ensure proper lysis of the cell suspension.
6. Separate the cell debris by centrifuging the lysate at 39,706× *g* at 4 °C for 20 min.
7. Quickly decant the supernatant into a fresh tube and proceed to metal affinity purification with this sample.
8. **OPTIONAL STEP** Filter the supernatant through a 0.22 μM syringe filter to ensure the removal of any cells from the pellet.

### 3.2. Metal Affinity Chromatography. Time for Completion: 2 h

1. Take 3 mL of Ni-NTA affinity resin suspension in a 20 mL gravity flow chromatography column.
2. Let the storage buffer flow through and wash the affinity resin thrice with 4 mL of ice-cold buffer LW.
3. Add the washed affinity resin (by suspending it in a small volume of buffer LW) to the cell lysis supernatant from step 3.1.
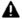
**CRITICAL STEP** Incubate this reaction tube in an end-over-end rotating shaker for exactly 15 min. As we do not add any protease inhibitors to the purification buffers, it is important to minimize the length of this incubation.
5. Load the suspension on a 20 mL gravity flow chromatography column and let the unbound fraction flow through.
6. Wash the resin thrice with 4 mL of ice-cold buffer LW.
7. Elute bound Cas9 from the column by adding ice-cold buffer EB (elution buffer) and collecting eight 1.5 mL fractions.
8. Estimate the concentration of protein in the elution fractions using an appropriate protein assay method (e.g., Bradford protein assay).
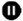
**OPTIONAL PAUSE STEP** The elution fractions with the highest protein concentrations can be pooled and dialyzed overnight against 1 liter of buffer A at 4 °C using a 50,000 Da or lower molecular weight cutoff membrane. While this step might serve as a pause point, it can be replaced by diluting the sample with buffer A or conducting buffer exchange using a centrifugal filter device to complete the entire protein purification within a day.

### 3.3. Cation Exchange Chromatography. Time to Completion: 1 h

1. **OPTIONAL STEP** The pooled elution fractions from step 3.2 can be concentrated and buffer exchanged into buffer A using a centrifugal filter. However, this optional step could add 30 min to the protocol.
2. For a chromatography system connected with a 10 mL sample injection loop, the pooled elution fractions can be diluted to 10 mL using buffer A. A two times dilution of the sample with buffer A was sufficient to enable its binding to the column.
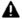
**CRITICAL STEP** Filter the sample by passing it through a 0.2 μM syringe filter before loading onto the chromatography column.
  NOTE: To save time and avoid degradation of protein, all the chromatography steps described below can be performed at the maximum permissible flow rate of 6 mL/minute for this column.
4. Inject the sample into a Resource S (6 mL) cation exchange chromatography column that is pre-equilibrated with 5 column volumes (CV) (30 mL) of buffer A.
5. Wash unbound proteins and buffer EB salts using 2 CV (12 mL) of buffer EB.
6. Elute bound proteins by increasing the volume of buffer B from 0 to 100% over 20 CV (120 mL). Collect 5 mL fractions of the elution.
7. Wash column with 5 CV of 100% buffer B followed by re-equilibration with 5 CV of 100% buffer A.
8. Estimate the concentration of protein in the elution peaks using the Bradford protein assay reagent.

### 3.5. Buffer Exchange and Protein Concentration. Time for Completion: 1 h

**Figure 2:**
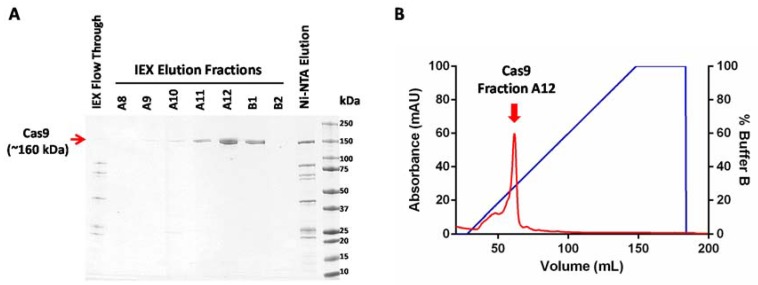
Purification of Cas9 protein. (**A**) SDS-PAGE (sodium dodecyl sulfate polyacrylamide gel electrophoresis) analysis of affinity enriched Cas9 fraction (Ni- NTA Elution) and ion exchange elution fractions A8 to B2 (IEX Elution Fractions). (**B**) Chromatogram showing elution of Cas9 protein from a cation exchange column. The elution fraction with the highest Cas9 content (Fraction A12) is highlighted by a red arrow.

1. Pool together fractions from the Cas9 elution peak (as indicated in Figure 2) and concentrate it using a centrifugal filter with a molecular weight cutoff limit of 50,000. The swinging bucket rotor can be set at a maximum of 4000× *g* for these filters and the samples can be spun for 5 min at 4 °C.
2. Once a roughly 10-times concentration is achieved, dilute the sample to the original volume with buffer A and centrifuge for a further 5 min.
3. Repeat this step at least three times to exchange the sample into buffer A.
4. Adjust the final volume of the sample according to the desired protein concentration for the downstream application.
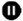
**PAUSE STEP** Add glycerol to the protein sample and aliquot into small usable fractions. Freeze samples with liquid nitrogen and store them at −80 °C. A final concentration of 5–20% glycerol can be used depending on the downstream application. These frozen samples can be stored for several months. However, repeated freeze–thaw cycles of the same fractions should be avoided.

## 4. Expected Results

**Figure 3:**
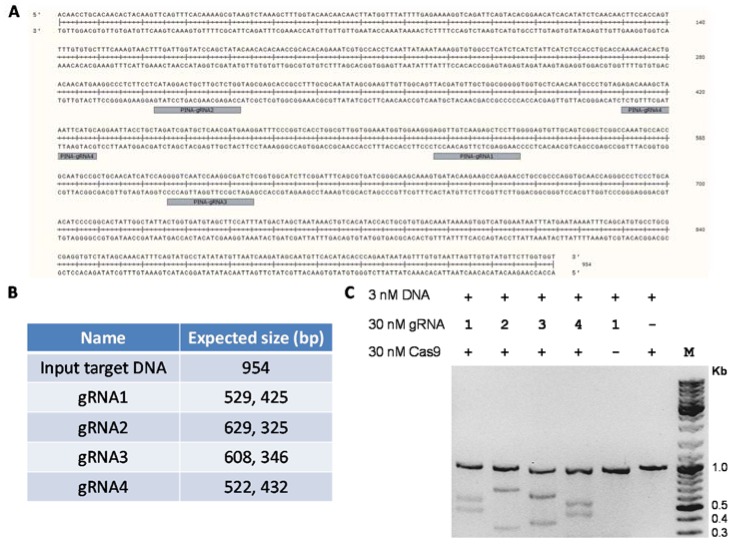
Functional validation of purified Cas9 protein by in vitro nuclease assay. (**A**) Sequence of the polymerase chain reaction (PCR)-amplified 954 bp DNA sequence of the wheat *pina* gene. The guide RNA (gRNA) target sites are highlighted. (**B**) The expected band sizes of the cleaved products using different guide RNAs against the 954 bp input DNA. (**C**) In vitro nuclease assay of Cas9 with different guide RNAs against the DNA target. Purified Cas9 endonuclease (30 nM), guide RNAs (30 nM) and PCR amplified target DNA fragment (3 nM) were mixed together at a molar ratio of 10:10:1 in a total assay reaction volume of 30 μL. The reactions were incubated at 37 °C for 15 min. The assay was stopped by the addition of 1 μL Proteinase K and products were analyzed using agarose gel electrophoresis.

Protein samples at every stage of purification were collected and analyzed using SDS-PAGE (sodium dodecyl sulfate polyacrylamide gel electrophoresis) (Figure 2A). A sharp peak containing pure Cas9 protein was observed in the cation exchange chromatogram. The protein yields at every step of purification are shown in Table 1. On average, this protocol yields approximately a milligram of highly pure Cas9 protein from a liter of recombinant *E. coli* culture. The enzymatic activity of Cas9 protein can be tested using an in vitro nuclease assay to further confirm the functionality of the purified protein (Figure 3). As the major contaminants from metal affinity enrichment do not bind to the cation exchange column and are found in the unbound flow through fraction (Figure 2A, lane 1), we speculate that this two-step purification protocol might work well for purification of other nuclease variants commonly used in genome editing, as long as their isoelectric point (pI) is high enough to enable them to interact with the cation exchange resin.

## 5. Reagents Setup

- Unless stated otherwise, all reagents were prepared with autoclaved dd H_2_O (henceforth, referred to as water)
- **1 M HEPES pH 7.5**. Dissolve 119.15 g of HEPES in 300 mL water, set pH with NaOH solution to 7.5, adjust final volume to 500 mL with water, filter sterilize through a 0.22 µM filter and store at room temperature for several weeks.
- **2 M KCl**. Dissolve 149.1 g of KCl in 1 L of water, sterilize by autoclaving and store at room temperature for several weeks.
- **1 M MgCl_2_**. Dissolve 50.8 g of MgCl_2_·6H_2_O in 250 mL water, sterilize by autoclaving and store at room temperature for several weeks.
- **3 M NaCl**. Dissolve 175.32 g of NaCl in 1 L of water, sterilize by autoclaving and store at room temperature for several weeks.
- **2.5 M Imidazole**. Dissolve 1.7 g of imidazole in 10 mL of water, filter sterilize through a 0.22 µM filter and store at 4 °C for a week.
- **0.5 M TCEP**. Dissolve 1.43 g of TCEP in 10 mL of water, filter sterilize through a 0.22 µM filter and store at 4 °C for several weeks.
- **Buffer LW** (for cell lysis, affinity column equilibration and washing unbound proteins).
  20 mM HEPES pH 7.5, 300 mM NaCl, 25 mM Imidazole, 0.5 mM TCEP. Mix together 2 mL of 1 M HEPES pH 7.5, 10 mL 3 M NaCl, 1 mL 2.5 M imidazole, 0.5 M TCEP, check pH and adjust to 7.5 using HCl, adjust final volume to 100 mL with water and sterilize by passing through a 0.22 μM filter and store at 4 °C for up to a week.
- **Buffer EB** (for elution from affinity column).
  20 mM HEPES pH 7.5, 300 mM NaCl, 250 mM Imidazole, 0.5 mM TCEP. Mix together 1 mL of 1 M HEPES pH 7.5, 5 mL of 3 M NaCl, 5 mL of 2.5 M imidazole, 0.05 mL of 0.5 M TCEP, check pH and adjust to 7.5 using HCl, adjust final volume to 50 mL with water and sterilize by passing through a 0.22 μM filter and store at 4 °C for up to a week.
- **Buffer A** (for cation exchange column equilibration and washing unbound proteins).
  20 mM HEPES pH 7.5, 200 mM KCl, 10 mM MgCl_2_, 0.5 mM TCEP. Mix together 8 mL of 1 M HEPES pH 7.5, 40 mL of 2 M KCl, 4 mL of 1 M MgCl_2_, 0.4 mL of 0.5 M TCEP, adjust final volume to 400 mL with water, sterilize and degas by passing through a 0.22 μM filter connected to a vacuum pump. Store at 4 °C for up to a week.
- **Buffer B** (for elution of proteins from cation exchange column).
  20 mM HEPES pH 7.5, 1 M KCl, 10 mM MgCl_2_, 0.5 mM TCEP. Mix together 4 mL of 1 M HEPES pH 7.5, 100 mL of 2 M KCl, 2 mL of 1 M MgCl_2_, 0.2 mL of 0.5 M TCEP, adjust final volume to 200 mL using water, sterilize and degas by passing through a 0.22 μM filter connected to a vacuum pump. Store at 4 °C for up to a week.

## Figures and Tables

**Table 1:** Protein yield at each step of Cas9 purification. The protein concentration, volume of sample obtained and amount of total protein in the sample were determined using the Bio-Rad protein assay reagent.

Sample Name	Concentration (mg/mL)	Volume (mL)	Total Protein (mg)
**Metal affinity enrichment**			
Ni-NTA Elution 1	5.9	1.5	8.85
Ni-NTA Elution 2	9.8	1.5	14.7
Ni-NTA Elution 3	1.2	1.5	1.8
Ni-NTA Elution 4	0.3	1.5	0.45
Ni-NTA Elution 5	0.2	1.5	0.3
Ni-NTA Elution 6	0.1	1.5	0.15
Ni-NTA Elution 7	0.1	1.5	0.15
Ni-NTA Elution 8	0	1.5	0
**Ni-NTA Total**			**26.4**
**Cation exchange chromatography**			
IEX A11	0.04	5	0.2
IEX A12	0.43	5	2.15
IEX B1	0.1	5	0.5
**IEX Total**			**2.85**
**Centrifugal filter buffer exchange and concentration**			
**Final Cas9 yield**	**2.4**	**1**	**2.4**
